# Development and Validation of an SNP-Based OpenArray^®^ Genotyping Panel for Discriminating *Coturnix coturnix*, *Coturnix japonica* and Their Hybrids

**DOI:** 10.3390/genes17070739

**Published:** 2026-06-26

**Authors:** Camilla Broggini, Alberto Membrillo, Javier Pérez-González, Romuald Rouger, Ines Sánchez-Donoso, Giovanni Vedel, Montserrat Nácher-Vázquez, José A. Torres, Eduardo Laguna, Celia Vinagre-Izquierdo, Jose Domingo Rodríguez-Teijeiro, Carles Vila, Juan Carranza

**Affiliations:** 1 Wildlife Research Unit (UIRCP-UCO), University of Cordoba, 14014 Cordoba, Spain; b72depoa@uco.es (A.M.); jcarranza@uco.es (J.C.); 2 Department of Specific Didactics, Faculty of Education Sciences, University of Cordoba, 14071 Cordoba, Spain; 3 Biology and Ethology Unit, Faculty of Veterinary, University of Extremadura, 10003 Caceres, Spain; 4 Syndicat des Sélectionneurs Avicoles et Aquacoles Français (SYSAAF), Centre INRAE Val-de-Loire, 37380 Nouzilly, France; romuald.rouger@inrae.fr; 5 Conservation and Evolutionary Genetics Group, Integrative Ecology Department, Estación Biológica de Doñana–Consejo Superior de Investigaciones Científicas (EBD-CSIC), 41092 Seville, Spain; 6 UIC Zoonosis y Enfermedades Emergentes ENZOEM, Universidad de Cordoba, Campus de Rabanales, 14014 Cordoba, Spain; 7 Department of Research, Fundación Artemisan, 13001 Ciudad Real, Spain; 8 Department of Animal Biology, University of Barcelona, 08028 Barcelona, Spain; jrodriguez@ub.edu

**Keywords:** common quail, *C. coturnix*, *C. japonica*, genetics, hybridization, OpenArray^®^

## Abstract

Background/Objectives: The common quail (*Coturnix coturnix*) is a game species facing conservation challenges, particularly hybridization with the Japanese quail (*Coturnix japonica*). To address this issue, one proposed measure is the urgent prohibition of releasing farmed quails into the wild. If authorized, mechanisms should be established to guarantee their genetic origin and prevent contamination of native populations. This work focuses on the development of a genetic tool based on Single-Nucleotide Polymorphism (SNP) markers that can differentiate between the two species and their hybrids. Our goal was to incorporate the selected markers into an OpenArray^®^ platform, to allow efficient, rapid, and cost-effective analysis. Methods: We tested two mitochondrial DNA SNPs (previously described in the literature) as diagnostic markers for species differentiation. We also assessed 24 nuclear DNA SNPs for compatibility with the OpenArray^®^ platform. Results: Of the 26 total SNPs, eight were excluded due to their limited utility. The remaining 18 SNPs achieved an overall genotyping success rate of 96.21%. Using the OpenArray^®^ platform with these 18 SNPs in a trial with samples from diverse Spanish field populations, we found 1.00% of *C. japonica* alleles (affecting 15.63% of individuals), suggesting introgression in the field. Population genetic analyses revealed strong differentiation between species and confirmed the presence of admixed individuals in field populations. Conclusions: This paper presents a new tool to differentiate between quail species and to identify foreign alleles in stocks and populations, by using an open platform system that optimizes the practical application of the diagnostic procedure based on the to-date most-reliable SNP markers for this goal.

## 1. Introduction

Phylogenetically related species, which normally maintain reproductive isolation in the wild, are increasingly experiencing genetic introgression due to anthropogenic interferences. Human activities—including habitat disturbance, introduction of non-native species, and climate change—create artificial contact zones between previously separated populations that break down reproductive barriers [1,2]. In this context, the release of gamebird stocks has become a major concern for the conservation of genetic integrity, as it can lead to the hybridization of wild populations with captive-bred individuals, causing loss of local adaptations and reduced genetic diversity [3].

The common quail (*Coturnix coturnix*) is a long-distance migratory species widely distributed across Europe and extending southward into North Africa; it occupies a broad range of habitats, including agricultural landscapes and grasslands [4,5].

The Japanese quail (*Coturnix japonica*), morphologically similar to the common quail, is native to East Asia, with a distribution across Russia, China, Mongolia, India, the Korean Peninsula, Japan, and Southeast Asia [6]. Outside its native range, it has been introduced into the United States, Italy, and Spain. The Japanese quail has been selectively bred in captivity for decades, mainly for meat consumption and egg production, commonly found in supermarkets [7,8]. Although the species is considered migratory, selective breeding in captivity has led to reduced dispersal ability and diminished antipredator behavior [9].

Although considered a common farmland species in Europe, the common quail faces several conservation threats, and its population is declining according to the International Union for Conservation of Nature (IUCN) [10]. Hunting bags in Spain, for instance, have decreased by nearly 50% over the past 20 years according to official statistics [11]. In many areas in Europe, quail populations in hunting grounds are often supplemented with farm-bred individuals [12]. This practice raises conservation concerns due to the risk of genetic introgression from the Japanese quail, which is frequently used in captive breeding. Captive-bred individuals are often released into the wild without prior verification of their genetic purity, representing a serious threat to the genetic integrity of wild common quail populations [13,14,15].

Genetically, *C. japonica* can hybridize with *C. coturnix*. Both *C. japonica* and its hybrids tend to perform better in captivity than *C. coturnix* [15], so crosses between female *C. japonica* and male *C. coturnix* are common among captive bred birds oriented to the release in hunting grounds [12,16].

In Spain, the release of farm-reared quails into the wild has been poorly controlled [12], with increasing sightings of *C. japonica* in the wild, in regions such as Castile and León, Madrid, and the Balearic Islands [17]. Although *C. japonica* and hybrids exhibit higher reproductive performance under captive conditions, they may be less adapted to natural environments due to altered behavioral traits [18]. As a result, individuals with low survival potential are introduced into natural habitats, leading to poor population replenishment and a continuous need for restocking. Ultimately, genetic introgression of *C. japonica* into *C. coturnix* populations may affect the adaptive potential of wild quail [13], raising serious conservation concerns.

A major concern with hybrid individuals is that even after several generations, they may carry genetic introgression from *C. japonica* while remaining morphologically indistinguishable from *C. coturnix*. For this reason, it is critical to develop reliable genetic and bioinformatic tools capable of detecting and differentiating hybrids and purebred individuals, thereby preventing their release into the wild [13].

In recent years, biotechnology and molecular biology have advanced significantly as tools to support decision-making in species conservation, taxonomic identification, and the selection of animal groups with higher productivity or desirable traits. Genetics, in particular, has emerged as a key method for identifying and selecting species and breeds, as well as detecting their hybrids, which were previously indistinguishable using traditional taxonomic approaches based on anatomical, morphological, physiological, and functional characteristics [19]. Molecular markers offer valuable tools for identifying individuals, populations, or species [20], as well as for detecting hybrids between species or divergent populations [21]. These tools have been employed in studies addressing hybridization between Italian grey partridge (*Perdix perdix*) and rock partridge (*Alectoris graeca*) [22], and between native red-legged partridge (*Alectoris rufa*) and invasive chukar partridge (*Alectoris chukar*) [23] in Spain.

Hybridization in fact has been widely documented in several *Phasianidae* species and may threaten the genetic integrity of native populations when non-native taxa are released [16,24]. In addition, mitochondrial heteroplasmy has been reported in different members of the family, including *A. graeca*, *A. chukar* and *P. perdix* [25,26], highlighting the need for multilocus approaches based primarily on nuclear markers when assessing introgression patterns.

To date, there has been limited progress towards establishing a standardized genetic control system for restocking practices involving quails, compared to, for example, the red-legged partridge [27].

Due to the phenotypic similarity between *C. coturnix* and its hybrids, there is a pressing need for public institutions, hunting grounds, and related organizations to collaborate on standardized mechanisms and certification methods to distinguish the species from its hybrids. Furthermore, public authorities must implement control measures to ensure compliance with this potential regulatory framework.

Prior to the publication of the Japanese quail reference genome in 2016 [28], microsatellite markers were the primary tools used to characterize the genetic origin of quail populations. Chazara et al. [14] used 25 microsatellite loci to assess genetic diversity. Sánchez-Donoso et al. [13] used a panel of microsatellite markers to characterize the genetic origin of quails, whereas Smith et al. [18] evaluated the genetic purity of a captive population of common quail using nuclear microsatellite markers.

Microsatellites are highly informative genetic markers with broad applicability. Although the use of a large number of microsatellite loci can provide substantial discriminatory power, previous results [29] highlight the need for further investigation of additional genetic markers to enhance genetic differentiation. In this context, classical assignment methods present an inherent limitation: any anonymous sample is necessarily assigned to one of the reference populations. Consequently, if admixed groups are not included among the reference populations, samples of mixed origin may be incorrectly classified [29]. Moreover, these methods are probabilistic, unlike Single-Nucleotide Polymorphisms (SNPs) which can serve as diagnostic markers under appropriate conditions [30]. The demonstrated effectiveness of SNP markers, together with the capacity to genotype large number of loci simultaneously through DNA chip technologies, has led to a transition from microsatellites to SNP-based approaches.

Previous studies in fact have identified diagnostic markers suitable for differentiating *C. coturnix* and *C. japonica*. In particular, Çeltik et al. (unpublished) identified a set of SNPs showing fixed allele frequencies in one of the two groups based on whole-genome sequencing. In addition, Rouger et al. [31] developed a panel of diagnostic SNP markers with high discriminatory power.

Mitochondrial DNA (mtDNA) has also been widely used in avian genetic studies. For instance, control region markers have been applied to assess genetic differentiation and hybridization in quail populations [21], while the *Cytochrome b* (*Cyt b*) gene has proven effectiveness for avian species identification [32].

Accordingly, the rationale for using SNPs in the present study is based on their suitability as diagnostic tools when they exhibit fixed or near-fixed allele frequency differences between populations [23]. In addition, their compatibility with automated genotyping platforms enables efficient and reproducible large-scale analyses. Among these, the OpenArray^®^ system (TaqMan OpenArray^®^ Genotyping System, Life Technologies^®^, Carlsbad, CA, USA) provides a flexible and cost-effective solution for implementing customized SNP panels with reduced sample and reagent requirements.

In this study, we developed and validated a panel of diagnostic SNPs to discriminate *C. coturnix*, *C. japonica*, and their hybrids, integrating these markers into an OpenArray^®^-based platform designed for routine application. This tool is intended to support conservation and management efforts by enabling reliable genetic identification and quality control of farm-reared and wild individuals.

## 2. Materials and Methods

### 2.1. Sample Collection and Processing

A total of 741 samples was collected between 2021 and 2025 from farms, supermarkets, zoos, and field areas with no evidence of captive quail releases across different parts of Spain. This study did not involve experiments on live animals. All samples were collected postmortem from quails legally hunted under local regulations and management plans; no animals were sacrificed specifically for this research. Accordingly, the ARRIVE guidelines are not applicable. DNA was extracted using the column-based protocol of the DNeasy Blood & Tissue Kit (Qiagen^®^, Hilden, Germany), following the manufacturer’s instructions. Samples consisted of fresh blood collected by a veterinarian and transported to the laboratory in EDTA tubes, as well as muscle tissue obtained immediately after legal harvesting and delivered promptly to the laboratory after the hunting activity. Once the DNA was extracted, concentration and quality parameters were analyzed by quantifying 2 µL of genomic DNA using a NanoDrop One spectrophotometer (Thermo Scientific, Waltham, MA, USA). Subsequently, DNA integrity was assessed through 0.75% agarose gel electrophoresis.

### 2.2. Primer Design and Thermochemical Protocols

For the selection of genes and markers relevant to the objective of this study, a comprehensive literature review was conducted to identify polymorphisms reported for differentiating *C. coturnix* and *C. japonica*. Based on previous studies, we selected 64 nuclear polymorphic loci of interest, including the 48 SNPs reported by Çeltik et al. (unpublished) and a subset of 16 markers from the panel described by Rouger et al. [31] chosen to minimize redundancy due to physical proximity among loci. In addition, two mitochondrial markers were included: one located in the control region [21] and another in the *Cyt b* gene [32].

Primer pairs flanking these loci were designed to amplify fragments containing the target polymorphism using NCBI Primer-BLAST (v2025) [33]. Regarding the fragment of the mitochondrial control region [21], the forward primer (PHDL) described by Randi and Lucchini [34] was used, whereas the reverse primer was redesigned to match the *C. japonica* sequence.

PCR amplifications were performed in a total reaction volume of 25 µL, containing 3 µL of genomic DNA, 1× PCR buffer, 0.75 µL MgCl_2_ (50 mM), 1.25 μL dNTPs (4 mM), 2.50 μL (5 μM) of each primer, and 0.10 μL (5U) of Taq DNA Polymerase (Biotools). Thermal cycling conditions consisted of an initial denaturation at 94 °C for 5 min, followed by 35 cycles of denaturation at 94 °C for 30 s, annealing at the optimal annealing temperature for 1 min, and extension at 72 °C for 1 min, with a final extension step at 72 °C for 10 min. Amplification was performed on 15 samples (5 *C. coturnix*, 5 *C. japonica*, and 5 hybrids), yielding 990 fragments in total. Amplicon integrity was verified by electrophoresis, and PCR products were visualized on a 1.5% agarose gel (Figure S1).

### 2.3. Sanger Sequencing

Following electrophoretic verification, PCR products were purified using ExoSAP-IT^™^ PCR Product Cleanup Reagent (Applied Biosystems, Waltham, MA, USA) to remove residual primers and unincorporated nucleotides prior to sequencing, and then re-visualized on an agarose gel to confirm product quality. Amplified fragments containing the target polymorphic loci were sequenced using an automated capillary sequencer (ABI 3130xl, Applied Biosystems, Foster City, CA, USA) at the Genomics Unit of the Central Research Support Service (SCAI), University of Córdoba and subsequently aligned and compared with the *C. japonica* reference sequence using Sequencher^®^ v.4.7 (Gene Codes Corporation, Ann Arbor, MI, USA) to identify polymorphic sites and determine the corresponding genotypes.

### 2.4. System Design for Target Polymorphisms

Once tested the 66 markers in 15 samples, 48 containing the polymorphisms described by Çeltik et al. (unpublished), 16 containing the SNPs reported by Rouger et al. [31], and 2 mitochondrial markers, 33 markers were selected based on their reported allele frequencies in the target species. These included 16 of the 48 markers from Çeltik et al. (unpublished), 15 of the 16 markers from Rouger et al. [31], and both mitochondrial markers.

Of these 33 markers, 7 were excluded for various reasons, including excessive physical proximity among candidate SNPs, poor sequence quality in the Sequencher^®^ project, the presence of a potential additional SNP within 1 bp of the target position, and ambiguous SNP signals that did not meet the reliability criteria for selection, resulting in a final panel of 26 SNPs (Table S1).

We subsequently validated the panel comprising 26 selected SNPs by direct sequencing of 44 samples (7 assumed *C. japonica*, 8 expected hybrids, and 29 probable *C. coturnix*) obtained from individuals of known origin, representing distinct production and ecological contexts. These included meat-production farms (putative *C. japonica*), farms breeding birds for release into hunting grounds (predominantly hybrids), and field-collected samples from areas with no evidence of restocking (likely *C. coturnix*).

### 2.5. OpenArray^®^ Genotyping Assays of Target Polymorphisms

For the analysis of these 26 SNPs, a medium-scale genotyping system was selected: the OpenArray^®^ open platform. The TaqMan^®^ OpenArray^®^ Genotyping Platform is a cost-effective, high-throughput technology based on real-time PCR for SNP analysis.

This system requires only small volumes of samples and reagents per reactions, combining high precision with operational simplicity. The platform offers flexibility by allowing a balance between the number of markers and the number of samples analyzed, as fewer markers permit the analysis of more samples, and vice versa.

The OpenArray^®^ genotyping technique is based on the hydrolysis of TaqMan^®^ probes. Each assay requires a pair of primers common to both wild-type and mutant sequences, together with two allele-specific probes: one targeting the reference sequence and labeled with the VIC^®^ fluorophore, and the other targeting the variant sequence and labeled with the FAM^®^ fluorophore (Table S2). Primers and probes are immobilized on microarray “spots” within a solid support matrix.

For this study, we selected a layout enabling the simultaneous analysis of 32 SNPs across 96 individuals. The number of markers was limited to 26, as six wells were reserved for internal quality controls. This list may be modified in the future, provided that any additional markers meet the same criteria regarding diagnostic performance.

### 2.6. Validation of the System Using Samples of Known Origin

After selecting the OpenArray^®^ plate layout, in order to validate the system, a study was conducted using the 26 main mutations associated with the hybridization between *C. coturnix* and *C. japonica* to test quail stocks for which we had previous information, allowing us to formulate hypotheses on their hybridization status. We included 741 samples: 534 from field populations, hence putative *C. coturnix,* and 119 putative *C. japonica* quails from farms. Along with these samples, we also included 88 putative hybrid quails. We also included 87 repeated samples as controls and technical replicates using the same sample at different DNA concentrations (Table 1).

To verify that the OpenArray^®^ system was reliably functioning, we carried out an additional refinement of the 26 selected markers, comparing the genotypes obtained through direct Sanger sequencing at the polymorphic sites of interest with those obtained via the OpenArray^®^ system, in 8 reference samples (putative hybrids). This comparison revealed discrepancies between the two methods in 8 of the markers. These markers were consequently excluded, reducing the final number to the 18 markers that produced consistent and concordant results across all cases.

### 2.7. Population Structure and Genetic Diversity Analyses

To describe the genetic structure of our samples based on the grouping of putative *C. coturnix*, putative *C. japonica* and putative hybrid individuals, we performed the following analyses. Genetic differentiation among groups was quantified using Weir and Cockerham’s FST, as implemented in the hierfstat package in R (4.5.1) [35], and genetic diversity was assessed through observed (Ho) and expected (He) heterozygosity. To obtain an assumption-free visualization of genetic structure and differentiation among individuals, principal component analysis (PCA) was performed in R using the adegenet package [36]. Missing data were replaced by mean allele frequencies. In this analysis, individuals that are closer in the multivariate space are genetically more similar. Population structure was further investigated using STRUCTURE v2.3.4 [37]. Ten independent runs were performed assuming K = 2, each consisting of 100,000 burn-in iterations followed by 100,000 Markov Chain Monte Carlo (MCMC) iterations under an admixture model. Because the aim of the analysis was to assess hybridization and introgression between the two parental species (*C. coturnix* and *C. japonica*), STRUCTURE was run assuming K = 2, corresponding to the two expected ancestral genetic groups, rather than to infer the optimal number of genetic clusters present in the dataset. The consistency among runs was assessed, and results were aligned using CLUMPAK web server (https://clumpak.evolseq.net/ accessed on 15 April 2026) [38]. Averaged membership coefficients were used for graphical representation to evaluate whether putative assignment based on origin was consistent with genetic composition.

## 3. Results

**Figure 1:**
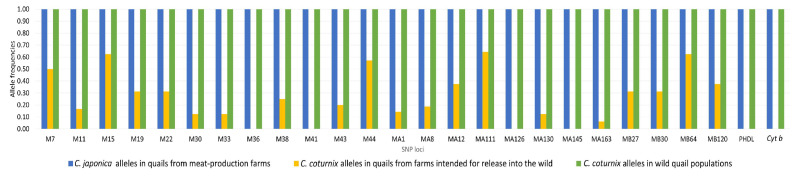
Allele frequencies of each of the 26 selected markers tested with the putative *Coturnix coturnix*, hybrids, and *Coturnix japonica* reference samples (*n* = 44).

To evaluate the diagnostic performance of the selected markers, we first assessed allele frequencies in the reference samples. The 26 SNP markers exhibited fixed allele frequencies (Table S3) across the 44 reference samples from meat-production farms and wild populations (Figure 1).

**Figure 2:**
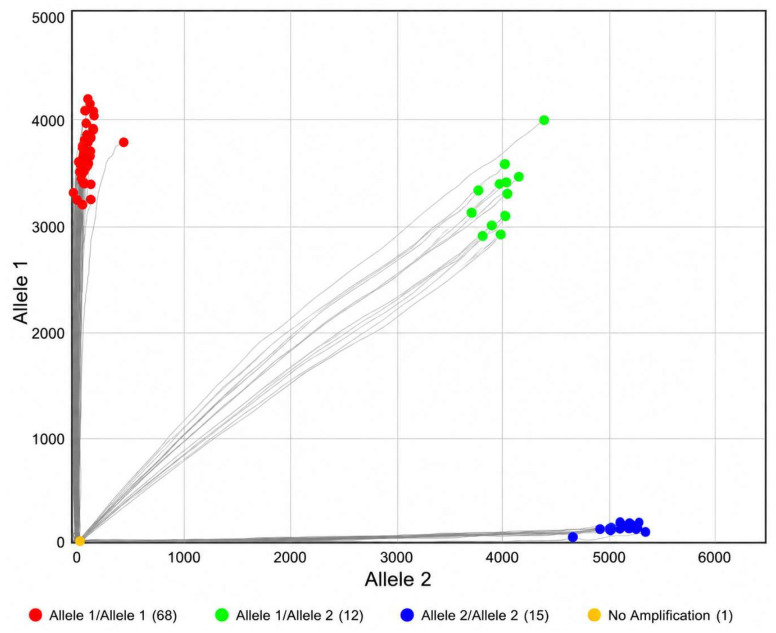
Scatter plot shows the mutation assay for the MB27 gene. Red dots represent samples with the wild *C. coturnix* sequence (allele 1) at both alleles. Green dots represent individuals carrying one *C. coturnix* allele and one mutant (*C. japonica*; allele 2) allele (heterozygous). Blue dots represent homozygous mutants carrying two copies of the mutant (*C. japonica*) allele. The yellow dot represents the negative control. The number of samples in each group is indicated in brackets. The axes show the fluorescence intensity of each color corresponding to the probes, measured in relative fluorescence units (RFU).

We used the designed system to analyze all 741 individual samples, along with 87 control samples, totaling 828 samples—equivalent to nine OpenArray^®^ plates, each configured to analyze 32 markers (26 SNPs and 6 internal controls, although in practice we used the 18 selected markers as explained above) across 96 individuals per plate. Following this, a total number of 21,528 (828 × 26) genotyping reactions were performed. As an example, Figure 2 shows a scatter plot with the outcomes for the MB27 marker. The three clusters generated, with *C. coturnix*, *C. japonica* and hybrid samples, allow the visual interpretation of the results.

For each individual quail sample, we recorded the number of markers and alleles successfully genotyped, as well as the number of *C. coturnix* and *C. japonica* alleles detected. From the 741 samples genotyped using the 18 final selected markers, 1001 out of 25,194 alleles could not be read. These failures were likely attributable to partial DNA degradation or low DNA concentration, and accounted for approximately 3.97% of the total alleles, which falls within the expected range for OpenArray-based assays [39].

**Figure 3:**
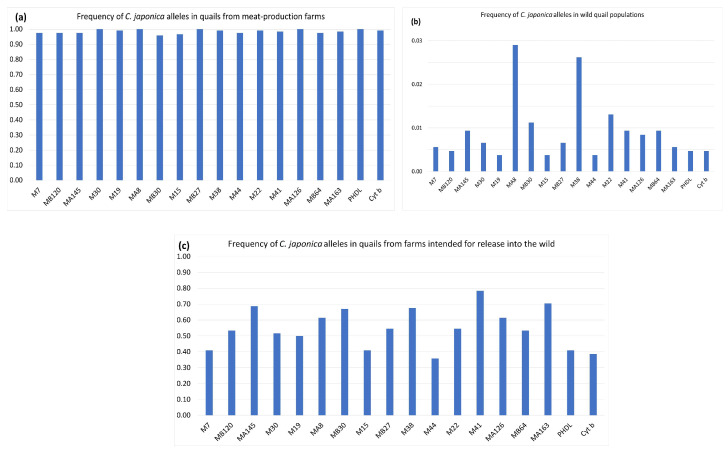
Frequencies of *C. japonica* alleles detected across the 18 analyzed markers (16 corresponding to nuclear loci and 2 to mitochondrial loci) in quails from meat-production farms (**a**), wild populations (**b**), and from farms intended for release into the wild (**c**).

Additionally, for each of the 18 markers analyzed, we calculated the total number and frequencies of *C. japonica* and *C. coturnix* alleles across the entire set of individual samples (Table S4). Thus, for quail samples from meat-production farms (*n* = 119), the mean frequency of *C. japonica* alleles across the set of 18 markers was 0.99. For wild population samples (*n* = 534), the mean *C. japonica* allele frequency was 0.01, while for hybrid individuals (*n* = 88), the mean frequency of *C. japonica* alleles was 0.55 (Figure 3).

Table 2 summarizes the genotyping results obtained from 534 putative *C. coturnix* individuals sampled across multiple field populations and analyzed using 18 SNP markers. Overall, 405 individuals showed no *C. japonica* mutant alleles, whereas 75 individuals carried one or more mutant alleles. 54 samples were classified as invalid due to amplification of fewer than 17 markers and hence were excluded from further analyses.

The proportion of individuals carrying one or more *C. japonica* alleles varied among sampling locations. The highest proportions were observed in La Rioja (4/10 valid samples, 40%), Soria (4/17, ~24%) and Seville (13/60 valid samples, 22%). In contrast, several populations exhibited low levels of introgression, with only one or two individuals carrying *C. japonica* alleles, such as those from Ciudad Real and Guadalajara.

When considering allele frequencies, the overall percentage of mutant alleles across all valid samples was 1.00% for the full marker set. The mean percentage at nuclear markers (16 loci) was 0.99%, while mitochondrial markers (2 loci) showed a slightly higher overall value (1.04%). Notably, mitochondrial mutant alleles were detected in only a few populations (Burgos, Huelva, and Teruel), while the remaining populations showed no evidence of mitochondrial introgression.

Overall, the results indicate a heterogeneous geographic distribution of *C. japonica* alleles across putative *C. coturnix* wild populations, with most individuals lacking detectable introgressed alleles, but a consistent minority showing evidence of hybridization, thus raising concerns about the occurrence of hybridization in the wild.

Pairwise FST values indicated strong genetic differentiation between putative *C. coturnix* and putative *C. japonica* (FST = 0.966), while putative hybrids showed intermediate differentiation, being closer to putative *C. japonica* (FST = 0.390) than to putative *C. coturnix* (FST = 0.802). Observed and expected heterozygosity were lowest in putative *C. coturnix* (Ho = 0.012, He = 0.019), intermediate in putative *C. japonica* (Ho = 0.136, He = 0.081), and highest in putative hybrids (Ho = 0.281, He = 0.452), consistent with admixed ancestry.

**Figure 4:**
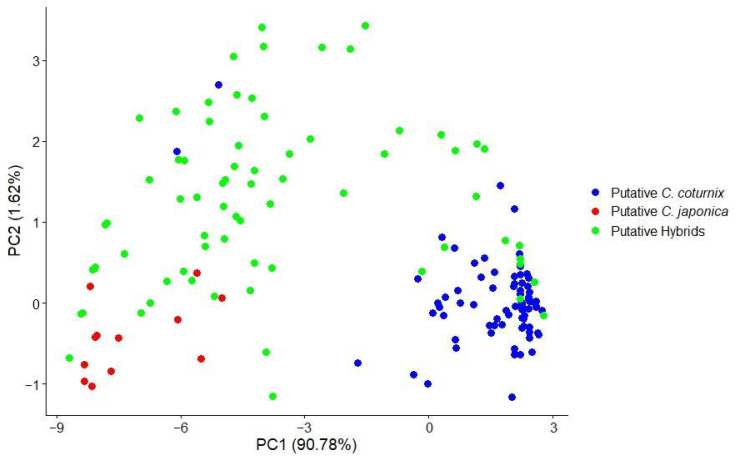
Principal component analysis (PCA) based on SNP genotypes showing genetic differentiation among individuals. Colors indicate initial categorization of individuals: putative *C. coturnix* (field samples), putative *C. japonica* (samples from meat farms, supermarkets and zoos) and putative hybrids (samples from hunting farms).

**Figure 5:**
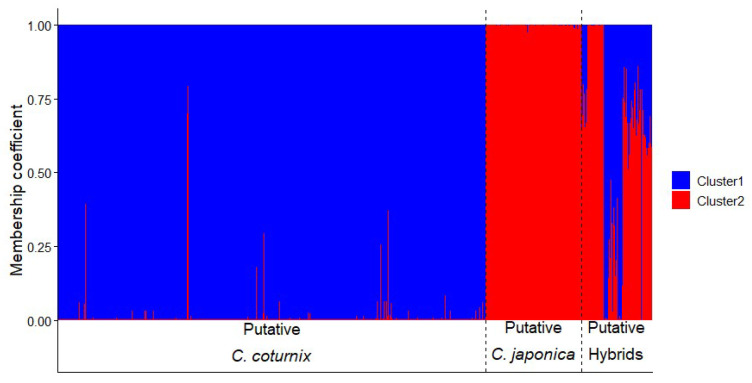
Bayesian clustering analysis performed in STRUCTURE (K = 2) based on SNP genotypes. Each vertical bar represents an individual, and colors indicate the proportion of membership to each genetic cluster. Individuals are grouped according to their initial assigned category: putative *C. coturnix* (field samples), putative *C. japonica* (samples from meat farms, supermarkets and zoos) and putative hybrids (samples from hunting farms).

PCA revealed a clear genetic differentiation between individuals classified as putative *C. coturnix* and putative *C. japonica*, supporting the general accuracy of their initial categorization. However, two field individuals originally assigned to *C. coturnix* based on their origin were positioned closer to the hybrid group in the multivariate space, suggesting a mixed genetic background. Hybrid individuals occupied intermediate positions along the axes, with some showing low genetic differentiation relative to the areas occupied by putative *C. coturnix* and putative *C. japonica* (Figure 4). STRUCTURE results were consistent with those obtained from PCA, with most putative *C. coturnix* and putative *C. japonica* individuals showing high membership coefficients to distinct genetic clusters (Figure 5). However, a few individuals initially classified as *C. coturnix* displayed elevated assignment probabilities to the cluster predominantly associated with *C. japonica*, indicating possible admixture. Similarly, putative hybrids exhibited mixed membership coefficients spanning the full range between both clusters.

## 4. Discussion

### 4.1. Technical Performance of the OpenArray^®^ System

The final panel of 18 diagnostic SNPs integrated into the OpenArray^®^ platform demonstrated excellent technical performance, achieving a global genotyping success rate of 96.21% across 741 analyzed samples (Table S5). This high reproducibility was confirmed by direct comparison with Sanger sequencing in reference samples, excluding eight inconsistent SNPs and retaining only those yielding 100% concordant results. Such success rates are comparable to or exceed those reported for SNP panels in similar mid-throughput platforms applied to game birds (e.g., 99.64% in red-legged partridge [23]; 74–91% in grouse (*Centrocercus* and *Tympanuchus* genera) [40]).

The platform’s flexibility, enabling analysis of up to 96 individuals per plate with 18–32 markers, optimally balances cost, sample volume, and throughput. This surpasses limitations of traditional microsatellite-based methods, which rely on probabilistic multi-locus assignments and are less amenable to automation.

### 4.2. Diagnostic Power and Marker Selection

Reduction from 66 initial SNPs to 26 (post-sequencing validation) and ultimately 18 (post-OpenArray^®^ calibration) prioritized markers with fixed allele frequencies between *C. coturnix* and *C. japonica*, primarily derived from Çeltik et al. (unpublished) and Rouger et al. [31]. The combined use of these 18 SNPs provides high statistical power to detect hybrid individuals, even at low levels of introgression. Assuming that the selected SNPs represent fixed differences between species and segregate independently, the probability of failing to detect introgression corresponds to the probability that all loci carry only *C. coturnix* alleles. Under this model, detection probability can be approximated as 1 − (1 − *p*)^n^, where *p* is the proportion of *C. japonica* ancestry and *n* is the number of diagnostic loci [41,42]. Based on this framework, detection probabilities are expected to be approximately 0.99, 0.91 and 0.69 for individuals carrying 25%, 12.5%, and 6.25% of *C. japonica* ancestry, respectively. These results indicate that early-generation hybrids are detected with high confidence, whereas detection power decreases for advanced backcrosses. Note that estimates assume independence among loci and complete diagnostic power of each SNP, conditions that may not be fully met in empirical datasets.

The theoretical expectations are consistent with the empirical performance of the SNP panel in our dataset. Individuals identified as hybrids based on the presence of *C. japonica* alleles showed admixed membership coefficients in STRUCTURE analyses and intermediate positions in PCA, whereas individuals lacking such alleles were predominantly assigned to the *C. coturnix* cluster [36,37]. This concordance between SNP-based classification and model-based clustering approaches provides empirical support for the diagnostic power of the marker panel.

The SNP panel presented here addresses key shortcomings of classical microsatellite assignment methods, such as forced allocation to reference populations and under-detection of admixed individuals, by providing deterministic discriminatory power. Validation in reference sample stocks confirmed 100% detection of *C. japonica* alleles in meat-production farms (*n* = 119, putative pure *C. japonica*) and, in the 88 release-farm individuals (expected hybrids), a mean *C. japonica* allele frequency of 0.55, consistent with the expected genetic composition of F1 hybrids, demonstrating diagnostic accuracy across reference categories. These results are in agreement with previous studies demonstrating the occurrence of hybridization and introgression in quail and other avian system [2].

The exclusions of markers due to physical proximity, sequence quality issues, or ambiguity ensured robustness of the final panel, as also highlighted in SNP-based studies of wildlife populations [43]. Although the current mid-scale panel provides high discriminatory power, the inclusion of additional loci could further improve the resolution for detecting low levels of introgression, as suggested in previous works [44].

### 4.3. Evidence of Introgression in Wild Populations

Application of the SNP panel to 534 field samples from areas with no evidence of recent releases revealed an overall frequency of 1.00% *C. japonica* mutant alleles (affecting 15.63% of individuals) across the 18 markers. Although allele frequencies were generally low (<2.00%), some geographic heterogeneity was observed, with relatively higher values in provinces such as Zaragoza (1.89%) and Teruel (1.29%), and lower values in areas such as Guadalajara (0.24%) and Badajoz (0.31%). These findings indicate ongoing introgression, consistent with prior records of *C. japonica* sightings in Castile and León, Madrid, and the Balearic Islands [17], as well as hybridization documented in Catalonia [45,46], and in west European countries [21].

Two hypotheses may explain *C. japonica* alleles in presumptively pure reference samples: technical failures or historical contamination of field quail populations. The latter is more plausible, considering system performance and evidence of unregulated releases, suggesting introgression may be more widespread than previously estimated using microsatellites [45].

Moreover, the detection of *C. japonica* alleles in the mitochondrial genome is particularly informative in support of the second explanation, as mitochondrial inheritance provides strong diagnostic confidence and cannot be explained by technical artifacts. The presence of *C. japonica* mitochondrial alleles in individuals sampled from wild populations indicates that introgression is occurring, or has occurred, even in natural environments. A plausible explanation for this pattern is the historical release of farm-reared quails into the wild, potentially dating back to periods prior to the implementation of regulatory measures aimed at preventing genetic introgression.

Such past releases may have resulted in long-lasting genetic signatures that are still detectable in contemporary wild populations. The samples with *C. japonica* mitochondrial alleles also carried *japonica* alleles at nuclear loci. The occurrence of *japonica* alleles at nuclear markers was not consistent across samples, as different loci were affected in different individuals rather than a single marker repeatedly showing introgression. This heterogeneous pattern, along with the concordance between mtDNA and nuclear markers in some samples, are unlikely to reflect a systematic technical artifact and instead supports the presence of introgressed or hybrid individuals within the field areas sampled.

### 4.4. Population Structure and Genetic Diversity

Our combined results from FST, heterozygosity estimates, PCA and STRUCTURE analyses consistently indicate a strong genetic differentiation between putative *C. coturnix* and putative *C. japonica*, supporting the effectiveness of the selected SNP panel for species discrimination. The high FST value and the clear separation observed in PCA and STRUCTURE confirm that the panel captures substantial genetic divergence between both *taxa*. Putative hybrid individuals showed intermediate genetic patterns, with higher heterozygosity and mixed assignment coefficients, consistent with admixed ancestry and varying degrees of introgression.

These population genetic analyses provide complementary evidence supporting the ability of the SNP panel to detect differentiation and identify admixed individuals. In particular, the detection of individuals whose genetic profiles did not match their origin-based classification highlights the limitations of relying solely on provenance information and reinforces the usefulness of the panel as a diagnostic tool for identifying individuals with mixed or unexpected genetic backgrounds. Importantly, these results are consistent with the main findings of the study and further support the presence of introgression in field populations.

The samples included in this study were selected to represent the main biological and management contexts relevant to the research question, including meat-production farms, release farms, and wild populations with no evidence of restocking. While broader geographic sampling could further improve the characterization of population structure, the current dataset provides a robust framework for evaluating the diagnostic performance of the SNP panel.

### 4.5. Implications for Conservation and Management

The SNP-OpenArray^®^ system presented here provides an operational tool for genetic certification: routine screening of breeders and release cohorts on farms, with strict thresholds (e.g., 0.00% *C. japonica* alleles for pure conservation stock). The reduced per-sample cost and high speed (96 samples/plate) render it feasible for regional authorities, hunting societies, and reference laboratories. It could prioritize reinforced controls and retrospective diagnostics of release policies, especially in regions with higher introgression risk, as well as preventive monitoring in all the natural distribution ranges of the species. This complements advances in red-legged partridge, fostering national standardization to safeguard *C. coturnix* genetic integrity, a species showing long-term declines in parts of its range, although populations along the western migratory route have remained relatively stable in the last decade [10,47]. This would not only help preserve the genetic integrity of *C. coturnix*, but also support the maintenance of certified game farms, which can provide quails for various purposes, including research and regulated sport hunting (e.g., dog training).

### 4.6. Limitations and Future Directions

Despite its strong performance, the current panel is limited by the number of diagnostic loci included. Although 18 SNPs provide substantial discriminatory power, the detection of advanced backcrosses remains incomplete, as predicted by theoretical expectations. The incorporation of additional markers from recently available genomic resources could further improve resolution.

Future work could also integrate model-based ancestry estimation approaches to quantify admixture proportions more precisely and assess temporal trends in introgression. The flexibility of the OpenArray^®^ platform facilitates such updates, making it a suitable framework for long-term monitoring and adaptive management strategies.

## 5. Conclusions

The OpenArray^®^ platform incorporating 18 diagnostic SNPs provides a robust, reproducible, and cost-effective mid-throughput tool for discriminating *C. coturnix*, *C. japonica*, and their hybrids in applied contexts. Field application revealed the presence of *C. japonica* alleles in farm stocks and in a subset of wild populations, indicating ongoing hybridization and introgression processes that may compromise the genetic integrity of the common quail. The high diagnostic performance of the SNP panel, including its ability to detect low levels of introgression, supports its use in genetic certification and release control programs aimed at minimizing further genetic contamination. The flexibility of the platform also allows future refinement through the incorporation of additional diagnostic markers, making it a suitable tool for long-term monitoring and management of quail populations.

## Figures and Tables

**Table 1:** Number of individuals in each sample subset and their origin.

Sample Subset	Number of Individuals	Origin
Field populations	534	Hunting areas in: Badajoz, Burgos, Cádiz, Canarias, Catalonia, Ciudad Real, Cordoba, Guadalajara, Huelva, Huesca, La Rioja, Lleida, Orense, Palencia, Seville, Soria, Teruel, Zamora, Zaragoza
Farm *C. japonica*	119	Farms and supermarkets in: Cordoba, Lleida and Zaragoza
Farm hybrids	88	Farms in: Badajoz and Sevilla

**Table 2:** Results for putative *C. coturnix* samples from field populations, analyzed with 18 SNP markers in the OpenArray platform. Total mutant alleles (%) were calculated across all 18 markers; Nuclear (%) across the 16 nuclear markers; mtDNA (%) across the 2 mitochondrial markers. The values reported in the last row correspond to the summed totals and overall frequencies calculated from all valid samples and markers.

Sample Sizes and Sources	Individuals with no *C. japonica* Alleles	Individuals with ≥1 *C. japonica* Allele	Invalid Samples (<17 Markers)	Total Mutant Alleles (%)	Nuclear Mutant Alleles (%)	mtDNA Mutant Alleles (%)
48/Badajoz	43	5	0	0.31	0.33	0.00
20/Burgos	15	4	1	7.89	7.73	10.53
15/Cádiz	11	3	1	0.63	0.67	0.00
4/Canarias	3	1	0	0.75	0.79	0.00
18/Catalonia	1	1	16	1.56	1.67	0.00
10/Ciudad Real	9	1	0	0.30	0.32	0.00
46/Cordoba	39	6	1	0.52	0.56	0.00
28/Guadalajara	23	2	3	0.24	0.26	0.00
37/Huelva	30	5	2	0.86	0.55	5.71
22/Huesca	14	3	5	0.52	0.56	0.00
10/La Rioja	6	4	0	1.47	1.56	0.00
25/Lleida	21	4	0	0.47	0.50	0.00
64/Orense	57	6	1	0.33	0.35	0.00
15/Palencia	12	3	0	0.99	1.05	0.00
72/Seville	47	13	12	0.70	0.75	0.00
20/Soria	13	4	3	0.70	0.75	0.00
38/Teruel	30	5	3	1.29	1.18	2.86
5/Zamora	0	0	5	0.00	0.00	0.00
37/Zaragoza	31	5	1	1.89	2.01	0.00
534/Total	405	75 (15.63%)	54	1.00	0.99	1.04

## Data Availability

The data supporting the results of this study are available within the article and its Supplementary Materials.

## Supplementary Materials

The following supporting information can be downloaded at: https://www.mdpi.com/article/10.3390/genes17070739/s1. Figure S1: Agarose gel electrophoresis showing PCR amplification products for the 18 selected SNP markers included in the OpenArray^®^ genotyping panel. Each panel corresponds to a different marker and includes five *C. coturnix* samples (Cc), five *C. japonica* samples (Cj), and five hybrid samples (HS). Technical replicates of selected hybrid samples are indicated as rHS. Fragment size and integrity were verified using a molecular weight marker (MWM; Dominion MBL^®^, 0–1000 bp). Negative controls (NTC) and blank wells (BLK), where applicable, are indicated in the corresponding gel panels; Table S1: Primers used for amplification of fragments containing the polymorphism of interest, amplicon sizes (bp—base pairs), SNP references and respective primers design [31,34]; Table S2: Primers and probes designed for the detection of each SNP; Table S3: Tabular summary of the results shown in Figure 1; Table S4: Tabular summary of the results shown in Figure 3; Table S5: Genotyping success rate.

## Abbreviations

The following abbreviations are used in this manuscript:

SNP	Single-Nucleotide Polymorphism
IUCN	International Union for Conservation of Nature
NGS	Next-Generation Sequencing
mtDNA	mitochondrial DNA
*Cyt b*	*Cytochrome b*
