# Supplementary material for: Development and Validation of an SNP-Based OpenArray® Genotyping Panel for Discriminating Coturnix coturnix, Coturnix japonica and Their Hybrids
Source: Genes (Basel). 2026 Jun 26;17(7):739. doi: 10.3390/genes17070739 (PMC13409662; doi:10.3390/genes17070739)
Supplement: Supplementary file 1 [file genes-17-00739-s001.zip › Rev_Supplementary_Figure_v4.pdf]

M7 (554)

MWM 1Cc 2Cc 3Cc 4Cc 5Cc 1Cj 2Cj 3Cj 4Cj 5Cj 1HS 2HS 3HS 4HS 5HS NTC BLK

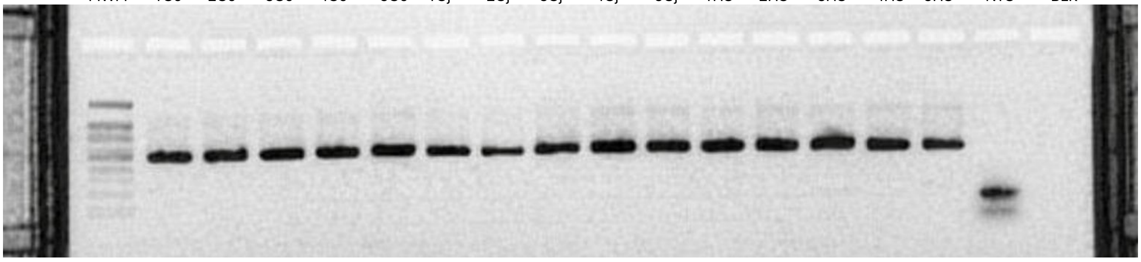

M15 (359)

MWM 1Cc 2Cc 3Cc 4Cc 5Cc 1Cj 2Cj 3Cj 4Cj 5Cj 1HS 2HS 3HS 4HS 5HS 2rHS MWM

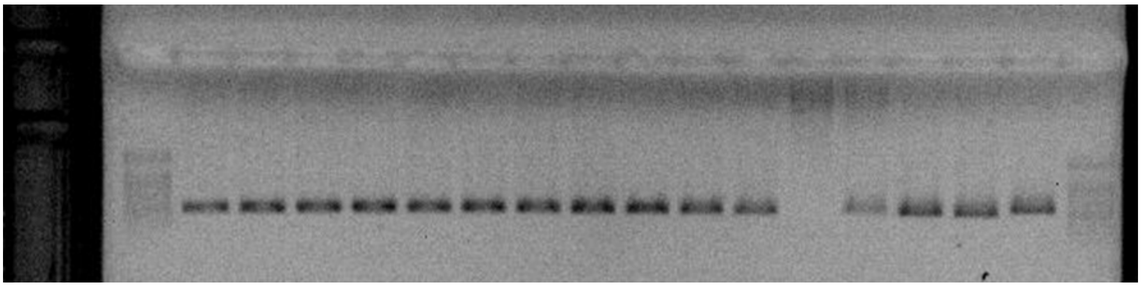

M19 (641)

MWM 1Cc 2Cc 3Cc 4Cc 5Cc 1Cj 2Cj 3Cj 4Cj 5Cj 1HS 2HS 3HS 4HS 5HS NTC BLK

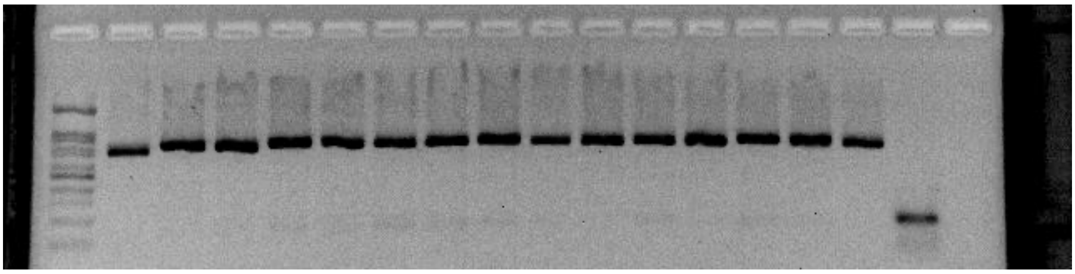

M22 (448)

MWM 1Cc 2Cc 3Cc 4Cc 5Cc 1Cj 2Cj 3Cj 4Cj 5Cj 1HS 2HS 3HS 4HS 5HS 2rHS 4rHS

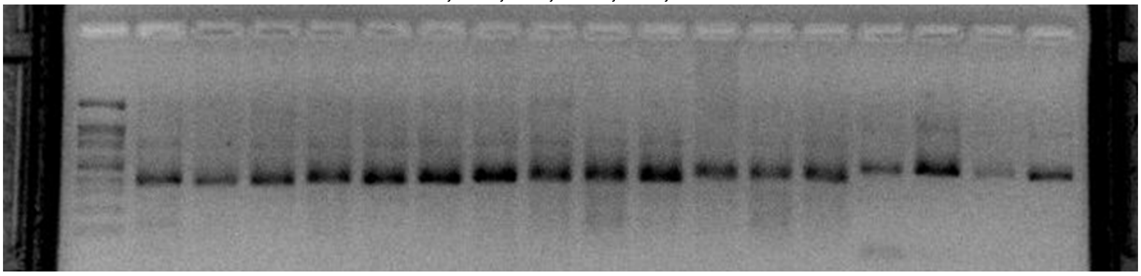

M30 (537)

MWM 1Cc 2Cc 3Cc 4Cc 5Cc 1Cj 2Cj 3Cj 4Cj 5Cj 1HS 2HS 3HS 4HS 5HS 3rHS BLK

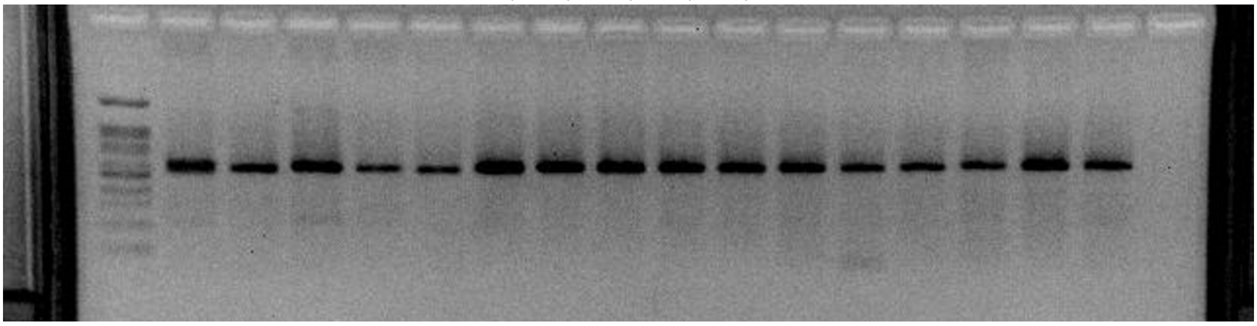

M38 (496)

MWM 1Cc 2Cc 3Cc 4Cc 5Cc 1Cj 2Cj 3Cj 4Cj 5Cj 1HS 2HS 3HS 4HS 5HS 1rHS BLK

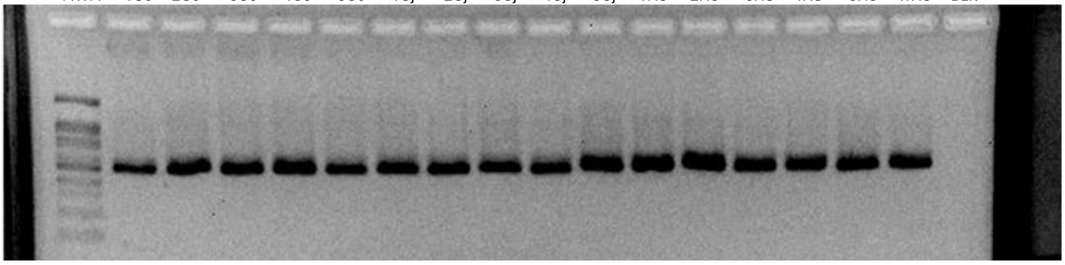

M41 (573)

MWM 1Cc 2Cc 3Cc 4Cc 5Cc 1Cj 2Cj 3Cj 4Cj 5Cj 1HS 2HS 3HS 4HS 5HS 2rHS 4rHS

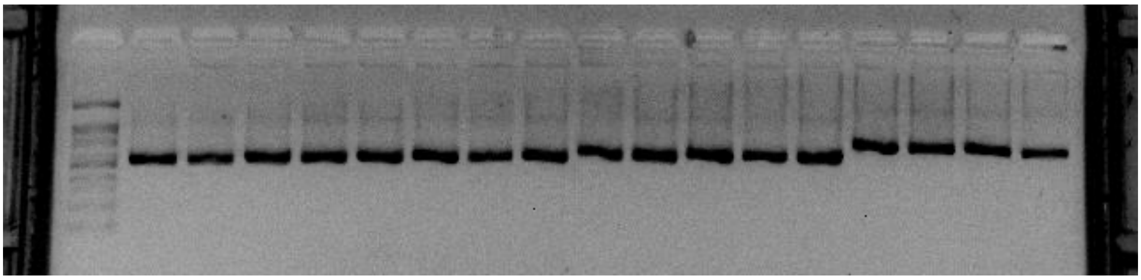

M44 (486)

MWM 1Cc 2Cc 3Cc 4Cc 5Cc 1Cj 2Cj 3Cj 4Cj 5Cj 1HS 2HS 3HS 4HS 5HS NTC BLK

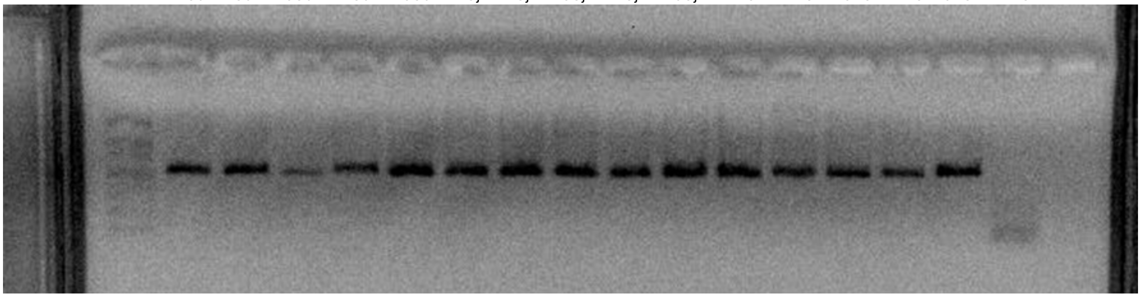

MA8 (570)

MWM 1Cc 2Cc 3Cc 4Cc 5Cc 1Cj 2Cj 3Cj 4Cj 5Cj 1HS 2HS 3HS 4HS 5HS NTC BLK

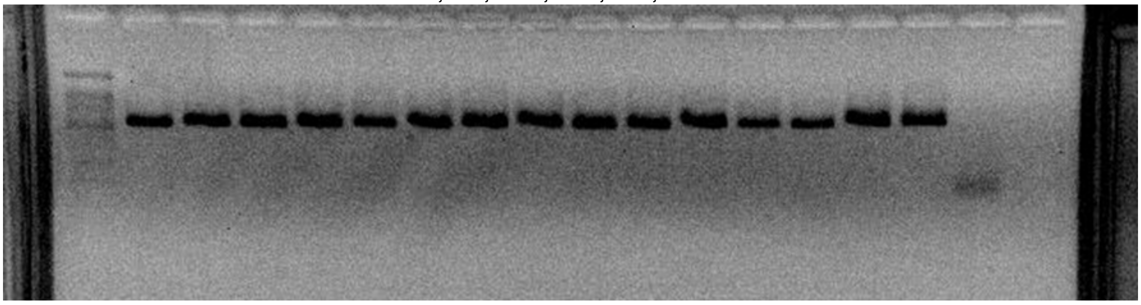

MA126 (635)

MWM 1Cc 2Cc 3Cc 4Cc 5Cc 1Cj 2Cj 3Cj 4Cj 5Cj 1HS 2HS 3HS 4HS 5HS 2rHS 5rHS

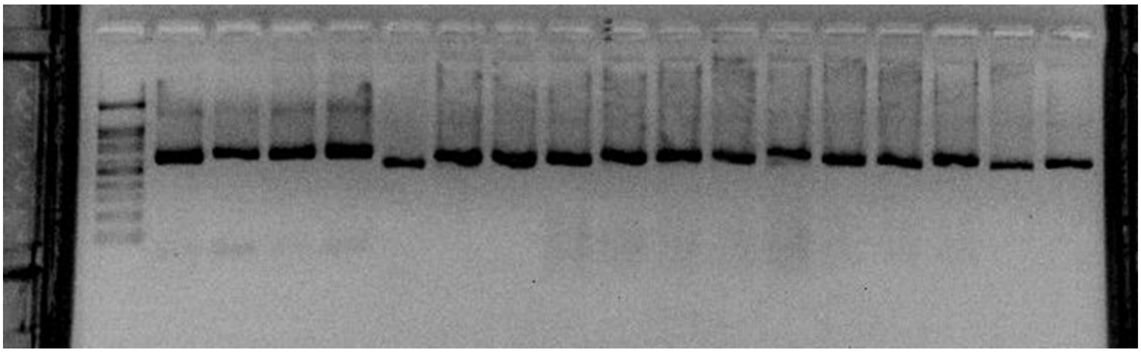

MA145 (501)

MWM 1Cc 2Cc 3Cc 4Cc 5Cc 1Cj 2Cj 3Cj 4Cj 5Cj 1HS 2HS 3HS 4HS 5HS 5rHS NTC

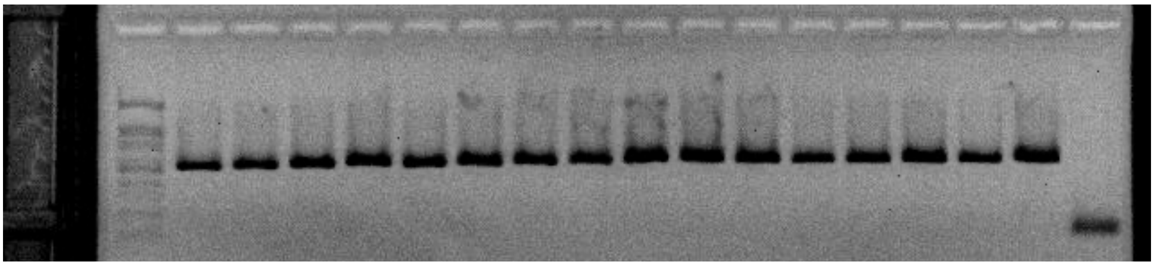

MA163 (561)

MWM 1Cc 2Cc 3Cc 4Cc 5Cc 1Cj 2Cj 3Cj 4Cj 5Cj 1HS 2HS 3HS 4HS 5HS 4rHS BLK

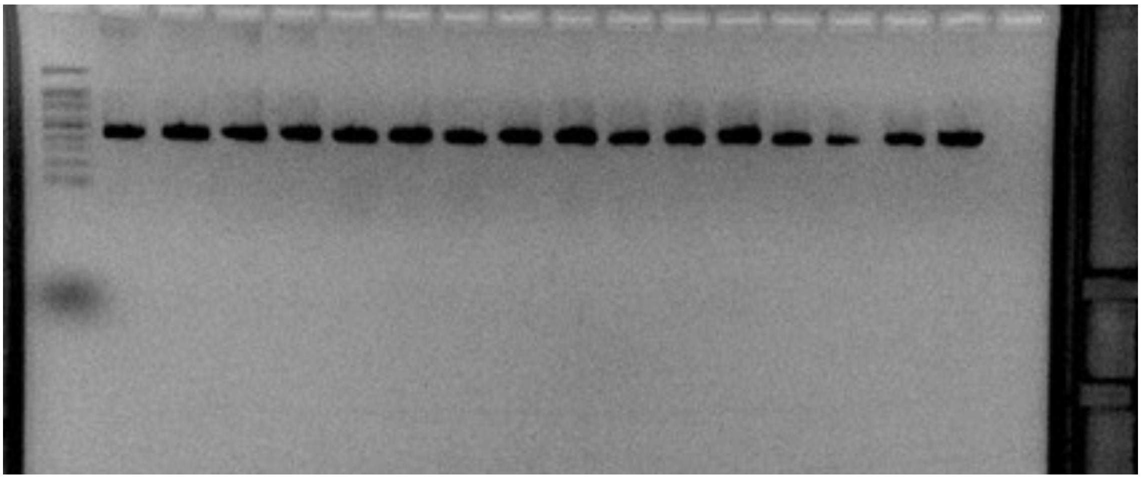

MB27 (510)

MWM 1Cc 2Cc 3Cc 4Cc 5Cc 1Cj 2Cj 3Cj 4Cj 5Cj 1HS 2HS 3HS 4HS 5HS NTC BLK

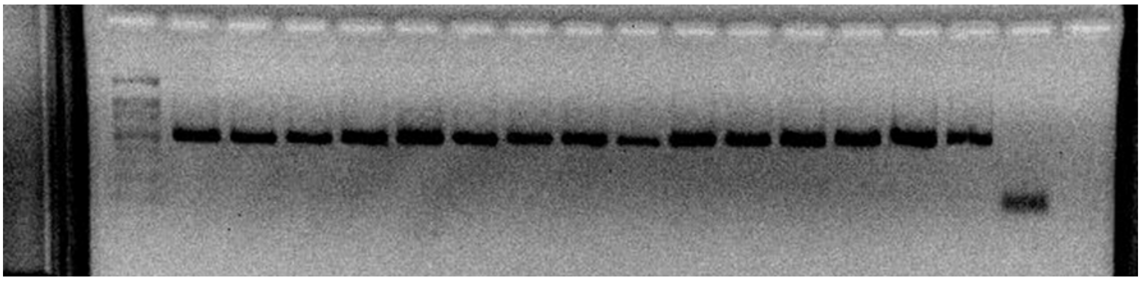

MB30 (529)

MWM 1Cc 2Cc 3Cc 4Cc 5Cc 1Cj 2Cj 3Cj 4Cj 5Cj 1HS 2HS 3HS 4HS 5HS BLK BLK

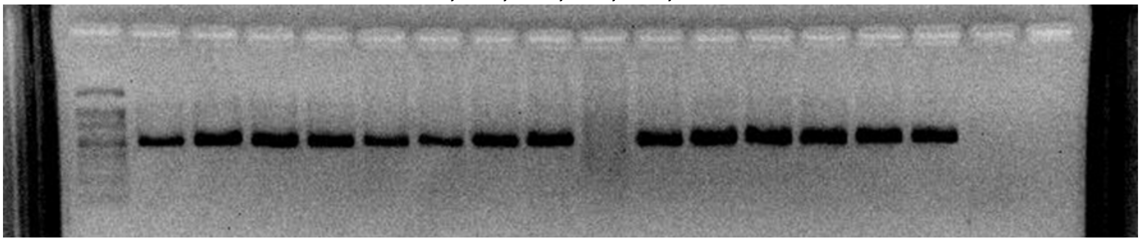

MB64 (760)

MWM 1Cc 2Cc 3Cc 4Cc 5Cc 1Cj 2Cj 3Cj 4Cj 5Cj 1HS 2HS 3HS 4HS 5HS NTC BLK

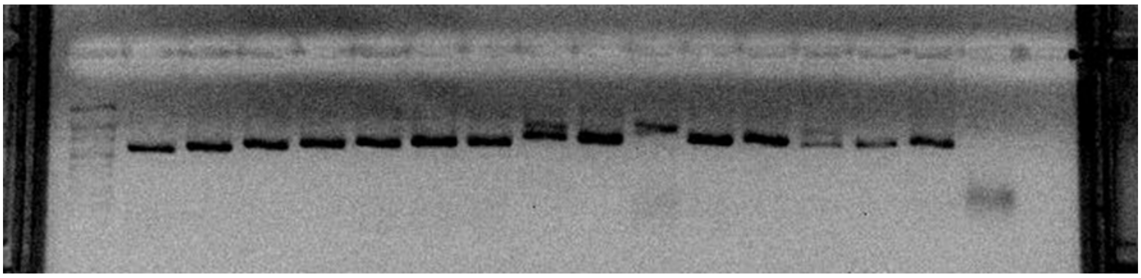

MB120 (562)

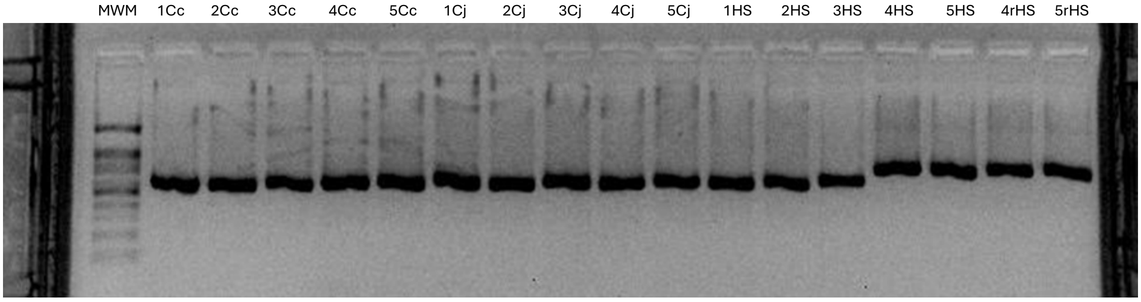

PHDL (527)

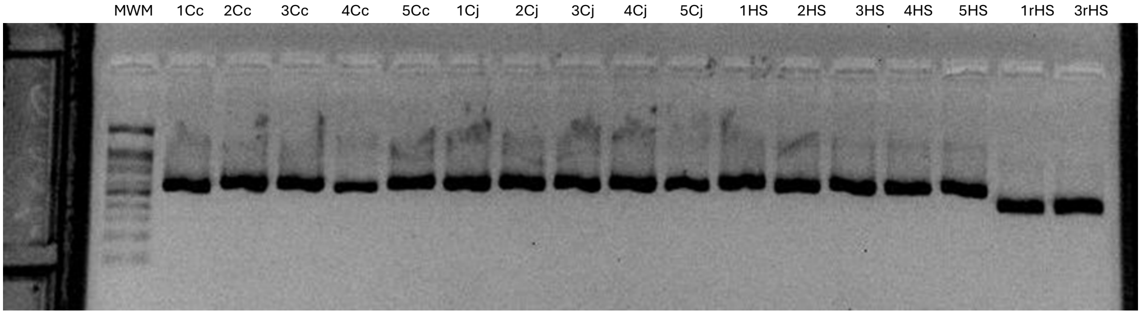

Cyt B (843)

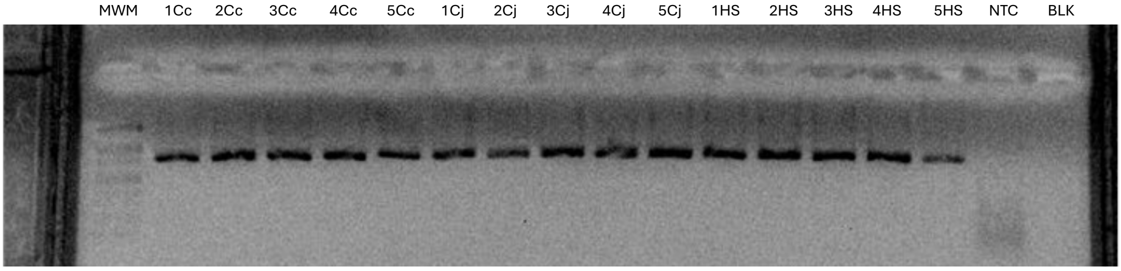

Figure S1. Agarose gel electrophoresis showing PCR amplification products for the 18 selected SNP markers included in the OpenArray® genotyping panel. Each panel corresponds to a different marker and includes five *Coturnix coturnix* samples (Cc), five *Coturnix japonica* samples (Cj), and five hybrid samples (HS). Technical replicates of selected hybrid samples are indicated as rHS. Fragment size and integrity were verified using a molecular weight marker (MWM; Dominion MBL®, 0–1000 bp). Negative controls (NTC) and blank wells (BLK), where applicable, are indicated in the corresponding gel panels.
